# Long-term exposure to high altitude reduces alpha and beta bands event-related desynchronization in a Go/NoGo task

**DOI:** 10.1038/s41598-023-45807-8

**Published:** 2023-11-13

**Authors:** Jianmin Hou, Cheng Wang, Lei Jia, Hailin Ma

**Affiliations:** 1 School of Psychology, Zhejiang Normal University, Jinhua, 321004 China; 2 Intelligent Laboratory of Child and Adolescent Mental Health and Crisis Intervention of Zhejiang Province, Zhejiang Normal University, Jinhua, Zhejiang China; 3 Plateau Brain Science Research Center, Tibet University, Lhasa, 850000 China

**Keywords:** Neuroscience, Cognitive neuroscience, Cognitive control

## Abstract

More than 80 million people worldwide permanently live at high altitudes, and living in such a hypoxic environment can impair cognitive functions. However, it is largely unknown how long-term exposure to high altitude affects neural oscillations underlying these cognitive functions. The present study employed a Go/NoGo task to investigate the effects of long-term exposure to high altitude on neural oscillations during cognitive control. We compared event-related spectral perturbations between the low-altitude and high-altitude groups, and the results revealed increased theta event-related synchronization (ERS) and decreased alpha and beta event-related desynchronizations (ERDs) during the NoGo condition compared to the Go condition. Importantly, the high-altitude group showed reduced alpha and beta ERDs compared to the low-altitude group, while the theta ERS was not affected by altitude. We suggest that long-term exposure to high altitude has an impact on top-down inhibitory control and movement preparation and execution in the Go/NoGo task.

## Introduction

Around the world, over 80 million individuals reside at altitudes above 2500 m above sea level^1^, while an even larger number of people visit high-altitude regions each year. Notably, the average altitude of the Qinghai-Tibet Plateau in China exceeds 4000 m, and this area is inhabited by more than 14 million permanent residents^2^. However, living at such high altitudes causes a decline in the partial pressure of inhaled oxygen, which in turn leads to a decrease in the hemoglobin oxygen saturation levels (%SpO_2_). Ultimately, these physiological changes can result in hypoxia^3,4^. Oxygen is essential to all humans, and hypoxia can cause physiological and cognitive impairments^5^. A growing body of evidence from both local residents and immigrants^6–8^ has shown the impact of exposure to high altitude on cognitive functions, such as attention^8^, memory^4^, and cognitive control^9^.

Cognitive control is a crucial skill that enables individuals to execute goal-directed actions, adapt to changing environments, and monitor and regulate behavior. It encompasses various cognitive processes such as response inhibition, response preparation, task switching, and updating of working memory^10^. Response inhibition, which refers to the ability to suppress reactions that are not currently needed or are inappropriate, is a core component of cognitive control^11,12^. The Go/NoGo task is a well-established paradigm for studying response inhibition^13,14^. In this task, participants are presented with a series of stimuli, a majority of which require a “Go” response and a minority that require a “NoGo” response. Thus, response inhibition is uniquely elicited in the NoGo condition^15^. Brain imaging studies have outlined the brain regions responsible for response inhibition. Functional magnetic resonance imaging (fMRI) studies have shown that activation of the dorsolateral and ventrolateral prefrontal cortex (PFC) and anterior cingulate cortex (ACC) is associated with response suppression in the Go/NoGo task^16^. Schulz et al.^17^ reported that children with impaired inhibition ability showed markedly greater activation in the left ACC, bilateral ventrolateral PFC, and left medial frontal gyrus. Importantly, it has been shown that long-term exposure to high altitude results in structural and functional abnormalities in brain regions related to response inhibition. For example, a positron emission tomography (PET) study reported decreased regional cerebral glucose metabolism in the frontal cortex following high-altitude exposure^18^. In an MRI study, Wang et al.^19^ found lower cerebral blood flow in the ACC in high-altitude Tibetans compared to lowland participants. Yan et al.^20^ observed reduced gray matter volume in the bilateral prefrontal cortex and prefrontal cortex in high-altitude residents. These studies suggest that long-term exposure to high-altitude environment can lead to various impairments in brain regions associated with response inhibition.

Event-related potential (ERP) studies have identified two distinct stages involved in response inhibition: conflict monitoring and conflict resolution^13,21^. In the NoGo trials, participants need to monitor the conflict between the prepotent Go response and the need to inhibit it (i.e., conflict monitoring), and subsequently resolve the detected conflicts by suppressing the prepotent response (i.e., conflict resolution). The N2 and P3 components have been consistently associated with these two stages of response inhibition. Specifically, NoGo trials elicit a larger N2 amplitude in the frontocentral areas compared to Go trials, suggesting N2 is related to the earlier conflict-monitoring stage of response inhibition^13^. Additionally, larger P3 responses are often observed in the central area during NoGo trials than during Go trials, reflecting the subsequent conflict resolution stage of response inhibition^21,22^. Ma et al.^9^ examined the impact of long-term exposure to high altitude on ERPs in the Go/NoGo task. They found that the high-altitude group had a delayed NoGo-N2 latency in the frontocentral area compared to the low-altitude group. Moreover, the high-altitude group showed larger N2 amplitudes and smaller P3 amplitudes in the frontal area for both the Go and NoGo conditions, compared to the low-altitude group. These results suggested that long-term exposure to high altitude affected both stages of response inhibition.

ERPs have provided valuable insights into the temporal dynamics of response inhibition; however, non-phase-locked activities are lost in ERPs due to averaging in the time domain^23^. Furthermore, ERPs cannot provide information about neural oscillations in specific frequency bands, which have been proven to be fruitful in understanding the cognitive neural mechanisms underlining different aspects of behavior and cognition^24–26^. To overcome these limitations, researchers have utilized event-related spectral perturbations (ERSPs) calculated through time–frequency analysis^27^. This technique allows for the differentiation between amplitude and phase information, enabling researchers to capture phase-locked and non-phase-locked neural activities (see^28^ for a detailed explanation of this technique) across multiple frequency bands. These neural oscillations in specific frequency bands, such as theta, alpha, and beta, have been found to play a crucial role in cognitive processes involved in the Go/NoGo task, including response inhibition, response preparation, monitoring, switching, and updating of working memory^29–31^.

Specifically, the Go/NoGo task commonly elicits in the frontocentral area increased theta band power relative to baseline (referred to as event-related synchronization, ERS)^11,32,33^ and decreased alpha and beta bands power relative to baseline (event-related desynchronization, ERD)^11,34,35^. Typically, larger theta ERS has been found in the NoGo condition compared to the Go condition, indicating that frontal theta ERS reflects inhibitory control^15,32,33,36^. On the other hand, significant alpha and beta ERDs are observed in both the Go and NoGo conditions^11,34,35^. Research has shown that alpha and beta ERDs in the frontocentral areas, especially the sensorimotor area, begin before voluntary movement, continue during movement, and end with a post-movement rebound of power above baseline^34,35,37,38^. These findings establish alpha and beta ERDs as reliable neural markers of movement preparation and execution^34^. In addition to movement preparation and execution, alpha oscillation has also been linked to inhibitory control^39,40^, as evidenced by studies demonstrating increased alpha power (i.e., smaller alpha ERD) when participants were asked to inhibit a memory^41^ or a motor movement^42,43^.

While previous studies have investigated the impact of long-term exposure to high altitude on response inhibition in terms of temporal dynamics, little is known about how this exposure affects neural oscillations in specific frequency bands during response inhibition. To address this issue, the current study aimed to examine the EEG data of two distinct groups of participants: one residing in a low-altitude region and the other in a high-altitude region. Participants performed the Go/NoGo task while their brain activity was recorded. ERSPs were then computed to assess the differences in response inhibition between the two groups. Specifically, the investigation focused on the theta, alpha, and beta frequency bands to gain insight into the neural mechanisms underlying response inhibition in the context of high-altitude exposure.

## Methods

### Participants

Forty college students participated in the current study. All of them were born and raised in a low-altitude location (< 1000 m). The twenty participants in the high-altitude group (10 male, 21.78 ± 1.41 years) were studying at Tibet University in Lhasa (altitude = 3650 m) and had lived at high altitude for the past three years. The twenty participants in the low-altitude group (10 male, 22.75 ± 1.08 years) were from universities in Beijing (altitude = 43.5 m) and had never been to a high-altitude area. All participants were neurologically healthy, right-handed, and had normal or corrected-to-normal vision. Participants gave informed consent before the experiment. This study conforms to the Declaration of Helsinki, and was approved by the Ethics Committee of the Institute of Psychology, Chinese Academy of Sciences (ethical number: XZDXLL2023096).

### Procedure

**Figure 1:**
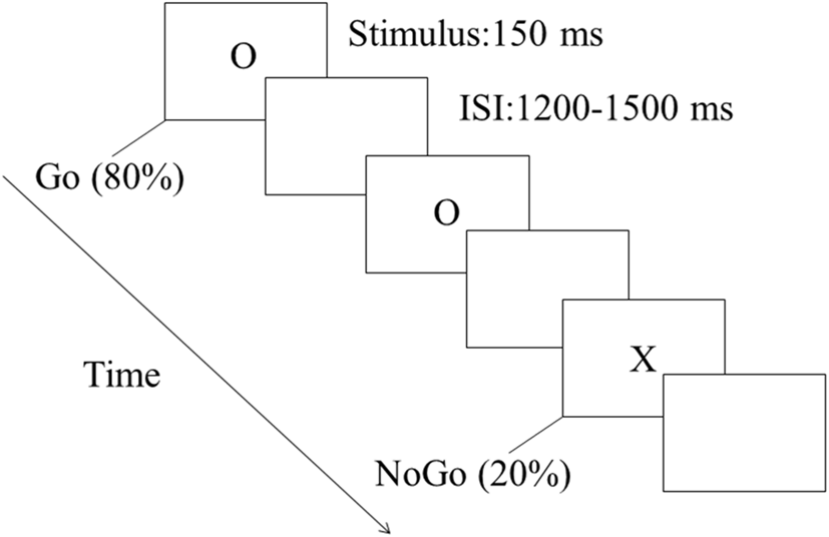
The Go/NoGo task diagram of experiment.

Participants were seated in a dimly illuminated, sound-attenuated room, where they completed a visual Go/NoGo task while EEG data was recorded. The task was divided into two blocks of 240 trials, with 192 Go trials (80%) and 48 NoGo trials in each block. Before the two experimental blocks, twenty practice trials were administered. The visual stimuli used in the experiment were two capital letters, 'O' and 'X', with one serving as the Go stimulus and the other as the NoGo stimulus. The associations between the letters ('O' and 'X') and the trial types (Go and NoGo) were counterbalanced across the blocks. Specifically, in one block, the letter 'O' was designated as the Go stimulus, while in the other block, it served as the NoGo stimulus. Each trial began with the presentation of 'O' or 'X' on the screen for 150 ms, followed by a blank as an interstimulus interval (ISI) ranging from 1200 to 1500 ms (see Fig. 1). The letters were displayed in white on a black background at the center of an LCD monitor (AOC 17-in) with a size of 2.6° × 1.8° of visual angle. Participants were instructed to respond with their right hand by pressing a key when presented with a Go stimulus and to withhold their response when presented with a NoGo stimulus. Both speed and accuracy were emphasized. As most of the trials were Go trials, the NoGo trials required participants to inhibit their response, thereby activating the response inhibition process. The E-prime software (﻿Version 1.1, Psychology Software Tools, Inc.) was used to present stimuli and collect behavioral data.

### EEG recording and preprocessing

EEG data were collected using a Neuroscan SynAmps amplifier from 64 scalp Ag/AgCl electrodes positioned according to the standard 10–20 scalp sites, with an elastic cap from Neuroscan Inc. An online reference electrode was placed approximately 2 cm posterior to CZ, while a ground electrode was placed between FPz and Fz. The electrooculogram (EOG) was recorded with bipolar montages placed above and below the left eye and on the outer canthi of both eyes to capture vertical and horizontal eye movements. All electrodes were maintained at an impedance below 5 kΩ. The EEG data were recorded continuously at a sampling rate of 500 Hz with a band-pass filter of 0.05–100 Hz.

The raw EEG data were preprocessed offline using the EEGLAB toolbox (version 2019)^44^ in MATLAB (The MathWorks, Inc., Natick, MA). The continuous EEG data was band-pass filtered (0.5–30 Hz), and visually inspected to remove segments containing large bursts of artifacts. Channels with poor signal quality were interpolated with the spherical method, and all channels were re-referenced to the average of the left and right mastoids. Epochs were extracted from −0.4 to 1 s relative to stimulus onset. Independent component analysis was applied to remove stereotypical artifacts such as eye movement, ECG and EMG, following the EEGLAB tutorial. Finally, epochs with amplitude exceeding ± 100 μV or containing artefacts were rejected, and trials with incorrect responses were excluded from further EEG analyses.

### Time frequency decomposition

ERSPs were calculated for EEG segments time locked to stimuli onset, ranging from 200 ms pre-stimulus to 800 ms post-stimulus onset, at frequencies ranging from 3 to 30 Hz in steps of 1 Hz, using a sliding time window of 400 ms in steps of 4 ms. EEG data in each of the sliding time window were multiplied with a Hanning taper, then Fourier transformed, resulting in a frequency-specific time course of complex numbers. Power was calculated as the squared norm of the complex numbers. Power was then averaged over trials per condition per participant, and normalized to mean power over baseline interval (−200 to 0 ms) using a decibel (dB) transform, which was done using the following formula: $10{\mathrm{log}}_{10}\left(\frac{power}{baseline}\right)$. Positive power relative to the baseline was referred to as ERS, with larger ERS indicating more positive power. Negative power relative to the baseline was referred to as ERD, with larger ERD indicating more negative power. These analyses were performed on the MATLAB platform using the Fieldtrip toolbox^45^ and custom-built scripts. The ERP analysis of this dataset was previously reported by Ma and colleagues^9^.

### Statistical analysis

**Figure 2:**
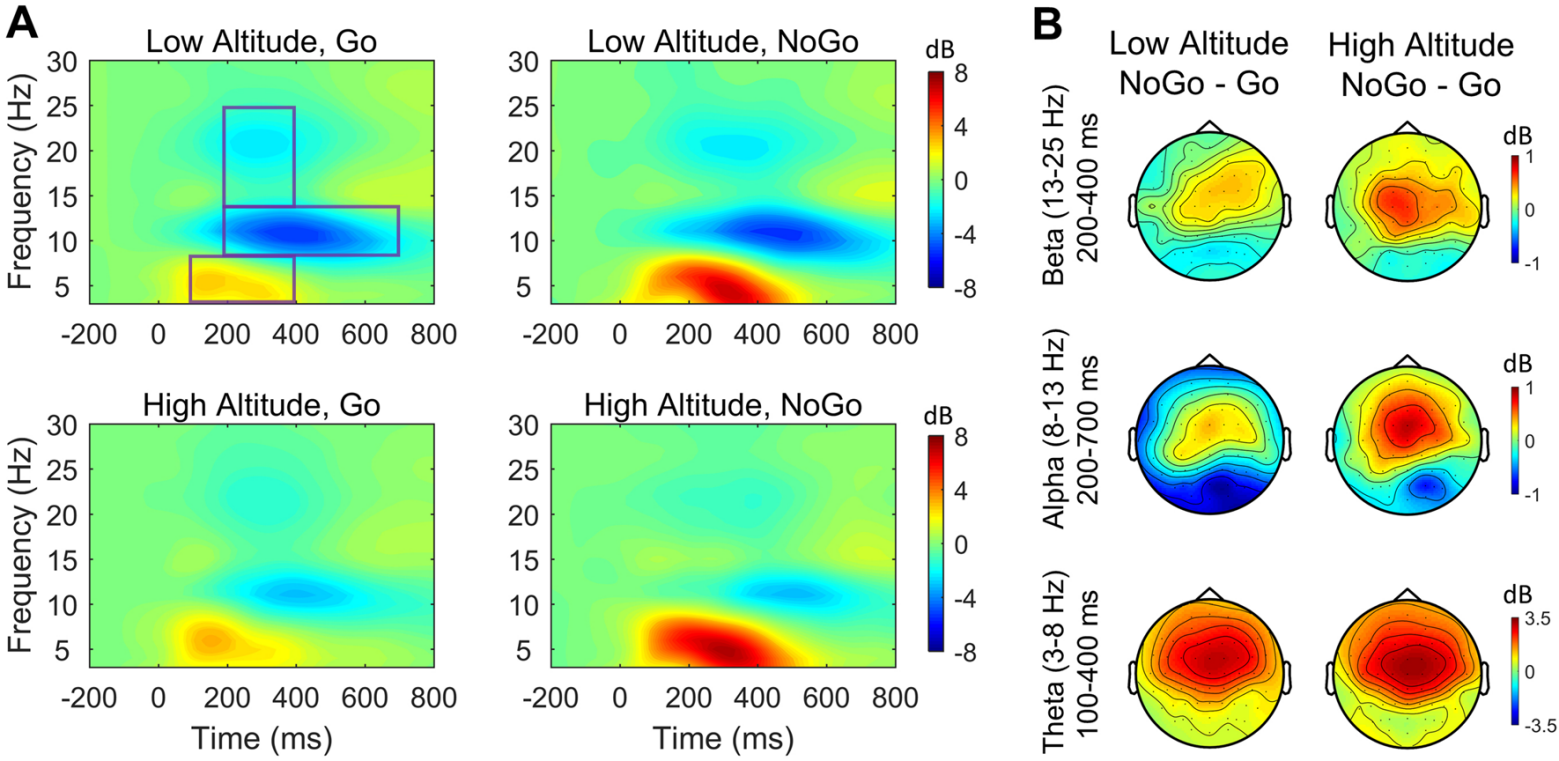
ERSP and topography of the Go vs. NoGo effect. (**A**) Grand averages of ERSP for each condition, averaged over the nine frontal-central electrodes (i.e., F1, FZ, F2, FC1, FCZ, FC2, C1, CZ, C2); enclosed by rectangles were the three time–frequency windows selected: theta band ERS (3–8 Hz) in the 100–400 ms time-window, alpha band ERD (8–13 Hz) in the 200–700 ms time-window, and Beta band ERD (13–25 Hz) in the 200–400 ms time-window. (**B**) Topographic plot of the Go vs. NoGo effect (NoGo minus Go) in each time–frequency window.

ERSP data were statistically analyzed within three frequency bands of interest (theta, alpha and beta) at the frontal-central midline electrodes (i.e., F1, FZ, F2, FC1, FCZ, FC2, C1, CZ, C2). These frequency bands were selected based on previous research which has shown that theta ERS and alpha and beta ERDs at the frontocentral areas are linked to response inhibition in the Go-NoGo task^11,32,33^. To best capture the ERS or ERD relative to baseline, specific time windows were chosen by visual inspection for each frequency band, as depicted in Fig. 2A. Thus, three time–frequency windows were selected: theta band (3–8 Hz) in the 100–400 ms time-window, and alpha band (8–13 Hz) in the 200–700 ms time-window, and beta band (13–25 Hz) in the 200–400 ms time-window, as shown by the three areas enclosed by rectangles in Fig. 2A. In each time–frequency-electrode window, power was averaged over time, frequency and electrode, then submitted to a mixed-design $2\times 2$ ANOVA with Group (low vs. high altitude) as a between-subject variable and Trial Type (Go vs. NoGo) as a within-subject variable.

## Results

**Figure 3:**
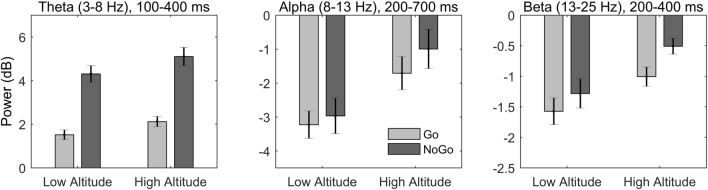
Mean power in each time–frequency window as a function of group and trial type.

Behavioral and ERP results were reported in a previously published study using the same EEG dataset^9^. Behavioral measures (including miss rate, false alarm rate, reaction time for correct responses, as well as *d’* and $\beta$ in signal detection analysis) showed no significant difference between the two groups. ERP results showed larger N2 and smaller P3 amplitudes in the high-altitude than the low-altitude groups for both Go and NoGo conditions (for details see^9^). The present study focused on the oscillatory activities associated with response inhibition and the effect of exposure to high altitude on them. The grand-average of ERSP for each condition was presented in Fig. 2A. Also see Fig. 3 for mean ERSPs averaged over the three time–frequency windows as a function of Group and Trial Type.

For the theta band (4–8 Hz) in the 100–400 ms time-window, the main effect of Trial Type was significant [$F\left(\mathrm{1,38}\right)=138, M{S}_{e}=1.21, p< 0.001, {\eta }_{p}^{2}= 0.784$], with larger ERS for NoGo trials ($\mathrm{M}=4.71,\mathrm{ SD}= 1.86$) than Go trials ($\mathrm{M}=1.81,\mathrm{ SD}= 1.09$). This effect was mainly distributed over the medial frontal-central part of the scalp (see Fig. 2B, bottom row). The main effect of Group was not significant ($p= 0.092$), and neither was the interaction between Trial Type and Group ($p= 0.700$).

For the alpha band (8–13 Hz) in the 200–700 ms time-window, the main effect of Trial Type was significant [$F\left(\mathrm{1,38}\right)=9.07, M{S}_{e}=0.53, p=0.005, {\eta }_{p}^{2}= .193$], with larger ERD for Go trials ($\mathrm{M}=-2.47,\mathrm{ SD}= 2.17$) than NoGo trials ($\mathrm{M}=-1.98,\mathrm{ SD}= 2.70$). This effect was mainly distributed over the medial frontal-central area (see Fig. 2B, middle row). The main effect of Group was also significant [$F\left(\mathrm{1,38}\right)=5.99, M{S}_{e}=10.16, p= 0.019, {\eta }_{p}^{2}= 0.136$], with larger ERD induced in the low-altitude ($\mathrm{M}=-3.10,\mathrm{ SD}= 2.13$) than the high-altitude ($\mathrm{M}=-1.35,\mathrm{ SD}= 2.46$) groups. The interaction between Trial Type and Group was not significant ($p= 0.167$).

For the beta band (13–25 Hz) in the 200–400 ms time-window, the main effect of Trial Type was significant [$F\left(\mathrm{1,38}\right)=16.81, M{S}_{e}=0.186, p<0.001, {\eta }_{p}^{2}= 0.307$], with larger ERD for Go trials ($\mathrm{M}=-1.29,\mathrm{ SD}= 0.91$) than NoGo trials ($\mathrm{M}=-0.89,\mathrm{ SD}= 0.96$). This effect was mainly distributed over the frontal-central area (see Fig. 2B, top row). The main effect of Group was also significant [$F\left(\mathrm{1,38}\right)=6.54, M{S}_{e}=1.38, p= 0.015, {\eta }_{p}^{2}= 0.147$], with larger ERD induced in the low-altitude ($\mathrm{M}=-1.43,\mathrm{ SD}= 1.05$) than the high-altitude ($\mathrm{M}=-0.76,\mathrm{ SD}= 0.71$) groups. The interaction between Trial Type and Group was not significant ($p= 0.290$).

## Discussion

To explore the effect of long-term exposure to high altitude on neural oscillations associated with response inhibition, the current study compared ERSPs elicited by a Go/NoGo task between two groups of participants: one residing in a low-altitude region (< 1000 m) and the other in a high-altitude region (3650 m). The findings of this study replicated the classic Go/NoGo effects, revealing a greater theta ERS and reduced alpha/beta ERDs during NoGo trials compared to Go trials. Importantly, the high-altitude group exhibited decreased alpha and beta ERDs relative to the low-altitude group, while theta ERS was unaffected by altitude. The interaction between Group and Trial Type was not significant. These results suggest that long-term exposure to high altitude affected top-down inhibitory control, as well as movement preparation and execution in the Go/NoGo task.

We observed clear theta ERS and alpha/beta ERDs shortly after stimulus onset, which was consistent with prior research^11,33,34^. We also found classic Go/NoGo effects on neural oscillations in the theta, alpha and beta frequency bands: theta ERS was larger in the NoGo condition compared to the Go condition, while alpha/beta ERDs were larger in the Go condition compared to the NoGo condition. In a Go/NoGo task, perceptual processing, attention, and the decision to inhibit or execute a motor action are involved, but inhibition is unique to the NoGo trials^15^, suggesting that response inhibition is responsible for the increased theta ERS^15,32,33,36^. Our finding of larger theta ERS in the NoGo condition aligns with the viewpoint that associates theta ERS with response inhibition. Several potential mechanisms may underlie this relationship. Theta oscillations play a crucial role in cognitive control processes. It is proposed that increased theta power reflects the activation of prefrontal cortical regions involved in monitoring and regulating behavior^29^. Supporting this, current findings show greater theta ERS during NoGo trials, indicating enhanced cognitive control during inhibitory responses. Moreover, theta oscillations contribute to the regulation of neural excitability, with increased theta power believed to exert inhibitory effects on neuronal populations, facilitating the suppression of unwanted motor responses^46^. This aligns with the observed greater theta ERS during NoGo trials, emphasizing the involvement of inhibitory mechanisms in response inhibition.

Alpha and beta ERDs are considered as neural markers for movement preparation and execution (Schmiedt-Fehr et al., 2016; Wu et al., 2019). In a Go/NoGo task, movement preparation occurred in both Go and NoGo trials but execution only happened in the Go trials. The average reaction time for Go trials in the current study was around 300 ms, which falls within the time window of the observed alpha/beta ERDs. Given the additional process of movement execution in the Go trials, it was unsurprising to observe larger alpha/beta ERDs in the Go conditions (also see Schmiedt-Fehr et al., 2016; Wu et al., 2019). Alpha oscillations have also been associated with top-down inhibitory control processes: an increase in alpha power has been observed during inhibition of a motion^42,43^ or a memory^41^, or while performing top-down control^47^. Inhibitory control could also be the mechanism behind our finding of smaller alpha ERD (i.e., larger alpha power) in the NoGo condition compared to the Go condition.

The main contribution of the current study to existing literature is including altitude as a factor in the Go/NoGo task and examining how exposure to high altitude affects neural oscillations during this task. Many studies have investigated the ERPs and neural oscillations in the Go/NoGo task^11,33,34^, and a few studies have explored how high-altitude exposure affects ERPs evoked by various cognitive tasks^9,48,49^. However, the influence of high-altitude exposure on neural oscillations induced by cognitive processing is largely unknown. The current study addresses this issue by using a Go/NoGo task, which is a classic paradigm for studying response inhibition and cognitive control. We found that alpha and beta ERDs were reduced in the high-altitude group relative to the low-altitude group for both Go and NoGo trials, while theta ERS was not affected by altitude. Since alpha/beta ERDs reflect movement preparation and execution and alpha ERD can also reflect inhibitory control, we suggest that long-term exposure to high altitude suppresses individuals’ abilities to prepare and execute movement and to exert top-down inhibitory control. One recent study^49^ reported similar reduced alpha and beta ERDs for high-altitude group, but using a mental rotation task.

Why does high-altitude exposure reduce alpha and beta ERDs? Alpha and beta ERDs are not only involved in movement preparation and execution but also are considered to represent cortical involvement^50^. Alpha and beta ERDs are enhanced when the brain is engaged in processing cognitive information or producing motor behavior^51^. Conversely, an increase in alpha or beta power (i.e., reduced ERDs) is believed to reflect cortical idling^37,52,53^. Consistent with this view, we observed a reduction in alpha and beta ERDs during the NoGo condition, which requires movement inhibition and potentially induces an "idling" state. Furthermore, a significant decrease in these ERDs was found in the high-altitude group compared to the low-altitude group. Participants in the high-altitude group resided at an elevation of 3650 m for three years and were tested in the same environment. The hypoxic conditions at such high altitudes are known to greatly reduce hemoglobin oxygen saturation^3,4^. Studies have shown that even a brief one-night exposure to such environment can reduce alpha and beta ERDs^51,54^. This is not surprising, given that the brain requires a substantial amount of oxygen to maintain its functioning (approximately 20% of the body's consumption). One possible strategy by which the brain copes with the low level of hemoglobin oxygen is by reducing its level of activation, thereby leading to reduced alpha and beta ERDs. Overall, the decrease in alpha and beta ERDs suggests reduced cortical involvement and potential impairment in cognitive processing during high-altitude exposure, which can be attributed to the decrease in oxygen availability at high altitudes.

While our study focused primarily on the impact of hypoxia on alpha and beta ERDs, there may be additional factors that contribute to the differences between the high-altitude and low-altitude groups. An alternative explanation could be that other lifestyle factors associated with living at high altitudes might influence these findings. Individuals living at higher altitudes may have different daily routines, engage in greater physical activity, and adhere to unique dietary patterns. These factors could potentially influence brain activity and contribute to the observed changes in alpha and beta ERDs. Additionally, variations in air pressure, temperature, and exposure to ultraviolet radiation at high altitudes may also impact cortical functioning. Further investigations are warranted to explore these alternative explanations.

In conclusion, the current study contributes to the existing literature on cognitive control by including altitude as a factor in the Go/NoGo task and investigating the impact of long-term exposure to high altitude on neural oscillations during this task. Our findings revealed typical Go/NoGo effects, with larger theta ERS and smaller alpha/beta ERDs for the NoGo trials compared to the Go trials. Importantly, we observed a reduction in alpha and beta ERDs in the high-altitude group. These results suggest that long-term exposure to high altitude affects top-down inhibitory control, as well as movement preparation and execution in the Go/NoGo task. Furthermore, our findings imply that the hypoxic conditions experienced at high altitudes may lead to diminished cortical involvement and potential impairment in cognitive processing.

## Data Availability

The datasets generated during and/or analyzed during the current study are available from the corresponding author on reasonable request.
